# Rapid Sequential Implication of the Human Medial Temporal Lobe in Memory Encoding and Recognition

**DOI:** 10.3389/fnbeh.2021.684647

**Published:** 2021-10-22

**Authors:** Domilė Tautvydaitė, Alexandra Adam-Darqué, Aurélie L. Manuel, Radek Ptak, Armin Schnider

**Affiliations:** Laboratory of Cognitive Neurorehabilitation, Division of Neurorehabilitation, Department of Clinical Neuroscience, University Hospital and University of Geneva, Geneva, Switzerland

**Keywords:** evoked potentials, medial temporal lobe, memory encoding, inverse solution, source memory, orbitofrontal cortex

## Abstract

The medial temporal lobe (MTL) is crucial for memory encoding and recognition. The time course of these processes is unknown. The present study juxtaposed encoding and recognition in a single paradigm. Twenty healthy subjects performed a continuous recognition task as brain activity was monitored with a high-density electroencephalography. The task presented New pictures thought to evoke encoding. The stimuli were then repeated up to 4 consecutive times to produce over-familiarity. These repeated stimuli served as “baseline” for comparison with the other stimuli. Stimuli later reappeared after 9–15 intervening items, presumably associated with new encoding and recognition. Encoding-related differences in evoked response potential amplitudes and in spatiotemporal analysis were observed at 145–300 ms, whereby source estimation indicated MTL and orbitofrontal activity from 145 to 205 ms. Recognition-related activity evoked by late repetitions occurred at 405–470 ms, implicating the MTL and neocortical structures. These findings indicate that encoding of information is initiated before it is recognized. The result helps to explain modifications of memories over time, including false memories, confabulation, and consolidation.

## Introduction

Memories undergo modifications over time, as they are rehearsed and re-encoded (Brainerd and Reyna, 2005; Schnider, 2018). This points to an intricate interplay between retrieval (recognition, recollection) and encoding of memories. These processes have distinct anatomical representations in the medial temporal lobe (MTL). The hippocampus is involved both in the encoding and retrieval of memories (Nadel and Moscovitch, 1997; Squire et al., 2004; Kim, 2015; Moscovitch et al., 2016), with the anterior part mediating encoding, the posterior part retrieval (Lepage et al., 1998; Zeineh et al., 2003; Kim, 2015). In contrast to the recollection of events, which invokes the hippocampus, recognition of familiarity appears to be dependent on the perirhinal cortex and to be possible without intervention of the hippocampus (Brown and Aggleton, 2001; Barbeau et al., 2011).

In contrast to the anatomical basis, little is known about the timing of this interplay between encoding and recognition, especially immediately after a stimulus has been presented and either induces the formation of a new memory or the evocation and re-encoding of an old memory. Based on previous studies (Schnider et al., 2002; James et al., 2009; Thézé et al., 2017; Raynal et al., 2020), we hypothesize that a new stimulus very rapidly (around 200 ms) evokes an encoding signal, which initiates the recording of any modulation of the activated memory trace, whose content is subsequently (>400 ms) consciously recognized. In this context, “encoding” denotes a signal or process stabilizing the memory trace representing a stimulus so that it can be recognized later on after intervening stimuli; “recognition” denotes a process that allows one to decide whether one has met a piece of information before or not; “memory trace” denotes the neural substrate of a memory.

The hypothesis is derived from studies with patients who, after orbitofrontal damage or disconnection, confuse reality, as evident in confabulations, act according to currently inappropriate memories (previous habits) and disorientation (Schnider, 2003, 2018). These patients specifically failed in a continuous recognition task (CRT) requiring the ability to distinguish between memories that pertain to current reality and memories that do not (Schnider and Ptak, 1999; Nahum et al., 2012). Correct performance of the task by healthy subjects evokes orbitofrontal activation, as seen with PET (Schnider et al., 2000), and a frontal positivity at 200–300 ms in evoked potentials (Schnider et al., 2002; Wahlen et al., 2011). Our hypothesis is that evoked memories (or thoughts) undergo the reality filtering process at 200–300 ms, before being recognized, but that this entire process –from evocation of a thought or memory to its recognition- is also being encoded (Schnider, 2018). This sequence –initiation of an encoding process, then modulation, then recognition of the thought or memory trace– would allow for later source monitoring, that is, the distinction between a memory that relates to a real event in the past as opposed to a pure thought (Johnson et al., 1993; Mitchell and Johnson, 2009; Thézé et al., 2017).

While no study has directly compared the evoked responses associated with encoding and recognition, the available evidence supports the idea that recognition occurs after memory encoding has been initiated. Novel stimuli elicit a positive fronto-central evoked response potential (ERP) at around 250–500 ms (Ranganath and Rainer, 2003; Patel and Azzam, 2005) which was referred to activity in the MTL (Ranganath and Rainer, 2003; Grunwald and Kurthen, 2006). We observed a positive frontal ERP at 200–300 ms upon immediate repetition of meaningful designs in a CRT (James et al., 2009). This potential emanated from the MTL (Nahum et al., 2011), had a memory protective effect (Thézé et al., 2016) and was absent in patients with focal MTL damage (Tautvydaitë et al., 2019). A recent study found that new items, compared with highly familiar fourth repetitions, evoked a positive frontal ERP at 200–300 ms, which was estimated to emanate from the MTL and suggested to reflect encoding (Raynal et al., 2020).

The timing of recognition has been derived from the observation of old/new effects. Familiarity with previously seen stimuli is typically represented in a midfrontal or central ERP at around 300–600 ms (Friedman, 1990; Schnider et al., 2002; Rugg and Curran, 2007; Addante et al., 2012). However, differentiation between correctly recognized and missed previously seen pictures has been described as early as 150–450 ms (Duarte et al., 2004). Recollection (remembering the specific stimulus with its context) is represented in a parietal potential at about 300–400 to 600 ms (Duarte et al., 2004; Rugg and Curran, 2007). Even though these studies used dissimilar paradigms, the data are compatible with the hypothesis that encoding is initiated before the recognition of information.

In this study we tested the hypothesis that recognition of meaningful visual stimuli as previously seen, or not, is preceded by an encoding signal. We designed a continuous recognition task (CRT) composed of easily identifiable line drawings which appeared several times: after initial presentation (“New” stimuli), they were immediately repeated up to 4 consecutive times and finally appeared again after 9–15 intervening stimuli. The idea was that, new stimuli in a CRT not only induce perceptual processing but definitely also undergo encoding. Similarly, stimuli repeated after 9–15 intervening items evoke an encoding process, as evident in better recognition of stimuli seen twice than stimuli seen only once (James et al., 2009). In contrast to their first appearance as “new” stimuli, they can now also be recognized as having been seen before. Thus, upon delayed repetition, these stimuli presumably undergo similar perceptual processing and encoding as when they appeared for the first time, but they additionally allow for recognition as previously seen. Stimuli overlearned through several repetitions were assumed not to evoke significant new encoding. They were used here as a “baseline activity” allowing to sort out processing steps common to their first appearance (“New”) and late repetition, i.e., presumably perceptual processing and encoding.

## Materials and Methods

### Participants

Twenty-three 18–35 years old healthy subjects with no history of neurological or psychiatric diseases participated in the study. Sample size was based on previous studies using similar evoked potential analyses with healthy subjects. Three subjects were excluded from analysis due to poor EEG signal. The retained 20 subjects (10 females) were 25.7 ± 3.8 years of age. Subjects gave a written informed consent to participate in the study, which was approved by the Ethical Committee of the Canton of Geneva. The study was conducted according to the Declaration of Helsinki.

### Paradigm

**FIGURE 1:**
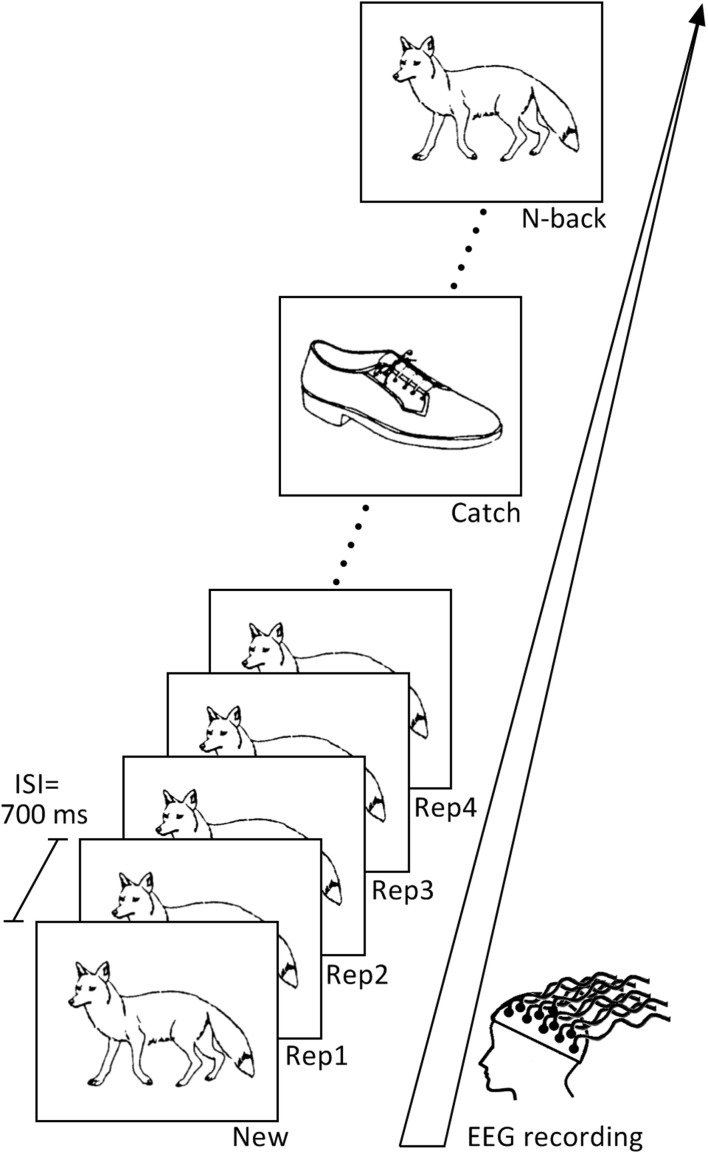
Continuous recognition task design. The learning task consisted of two blocks containing different set of pictures. Subjects had to indicate if the presented picture is new (New) or repeated. Pictures were repeated up to four consecutive times (Rep1, Rep2, Rep3, Rep4) and then re-appeared after 9–15 intervening items (N-Back). Pictures presented only once in the sequence (Catch) were inserted in order to avoid the habituation to the sequence.

We devised a continuous recognition task (CRT) composed of 72 pictures from Snodgrass and Vanderwart (1980) (Figure 1), a set of easily identifiable line drawings covering living and non-living categories. The list of stimuli presented in this task with their names and their ID numbers, as well as the code of experimental sequence protocol can be found on the data repository.^1^

After initial presentation as New items (*N* = 72), pictures were immediately repeated 3 times (Rep1, Rep2, Rep3 trials, *N* = 72), half of them even a fourth time (Rep4, *N* = 36) to preserve some variation within the task and avoid automatic responses. Stimuli then re-appeared again after 9–15 intervening items (N-back; *N* = 66; the 6 items at the end of the task were not repeated). To vary task sequence and sustain participants’ attention during the task, we also included pictures, which appeared only once (Catch trials, *N* = 22); they were presented randomly after Rep3, Rep4 or N-back stimuli. Subjects were instructed to press the “yes” button on the right side of a response box with their right middle finger if they had already seen the picture appearing on the screen, and the “no” button on the left side of the response box with their index finger if they thought they had not seen the stimulus before. Stimuli were presented on a white computer screen for 2,000 ms, with a 700 ms inter-stimulus interval.

To prevent fatigue, the task was divided into two equivalent parts composed of two different picture sets and separated by a 5 min’ break.

In order to test long-term retention, subjects passed a delayed recognition task 30 min after termination of the continuous recognition task. The test contained 60 pictures that had been repeated within the main task, 22 stimuli presented only once (Catch trials), and 30 novel pictures that the participants had not seen before. They were asked to indicate if the presented picture was new or previously seen in the preceding task. The list of items presented in delayed recognition task is provided on the data repository (see text footnote 1).

### Assumptions and General Analysis Strategy

The continuous recognition task (CRT) was designed to disentangle encoding and recognition by comparing New, N-back, and Rep3 stimuli. The following assumptions were made:

Stimuli appearing for the first time (New) undergo encoding, as reflected in their subsequent recognition both within the task after several intervening stimuli (Schnider et al., 2002; Wahlen et al., 2011) and after completion of the task (James et al., 2009). As newly presented stimuli always differ from the previous stimulus, the electrophysiological response presumably also reflects perceptual and possibly attentional processing.

Stimuli re-appearing after intervening stimuli (N-back) can be recognized as previously seen. Similar to their first appearance as New items, they are always preceded by a different picture. Thus, their processing differs from their first appearance as New by recognition, while perceptual and attentional challenges are probably similar. We thus interpreted differences in the electrophysiological response to N-back and New as reflecting recognition.

N-back stimuli are later on (typically in a delayed recognition task) better recognized than stimuli seen only once (e.g., Van Strien et al., 2007; James et al., 2009) or stimuli that are immediately repeated (James et al., 2009; Nahum et al., 2015), indicating that they also undergo encoding, similar to their first appearance as New. Thus, in order to sort out encoding processes presumably common to both New and N-back stimuli, we needed a comparison or “baseline” stimulus. For this purpose, we used the following stimuli:

Stimuli that are repeated after short lags or even several times in succession become over-familiar and are processed with less neural resources and faster, a processing difference known as repetition suppression (Brozinsky et al., 2005; Grill-Spector et al., 2006; Johnson et al., 2008). We thus presented stimuli repeatedly up to 4 consecutive times to use them as a “baseline,” assuming that on the third or fourth repetition, they no longer induce encoding. For analysis, we retained the stimuli repeated 3 times (Rep3).

Under these assumptions, encoding, and recognition were explored using the following comparisons:

Encoding was explored by independent comparison of New vs. Rep3 and N-back vs. Rep3 stimuli. Commonalities between these two contrasts were assumed to reflect encoding. As Rep3 stimuli have been preceded by themselves (as Rep2), their comparison with New and N-back trials possibly also encompasses differences in perceptual and attentional processing.

Recognition was explored by directly comparing responses in N-back and New trials.

### Electroencephalogram Acquisition and Preprocessing

Electroencephalogram (EEG) was recorded continuously during the learning task at 512 Hz using the 128 channels Active-Two Biosemi EEG system (BioSemi Active-Two, V.O.F., Amsterdam, The Netherlands). EEG data preprocessing and analyses were performed using the Cartool software developed by Denis Brunet^2^ (Brunet et al., 2011), and the Statistical Toolbox for Electrical Neuroimaging (STEN), developed by Jean-François Knebel and Michael Notter.^3^ To compute event related potentials (ERPs) epochs from 50 ms before stimulus onset to 550 ms post-stimulus onset were averaged for each subject and stimulus type of interest (New, Rep3, N-back). Only trials with correct responses were retained for analyses. Additionally to an automated ± 100 μV artifact rejection criterion, each trial was visually inspected to exclude epochs containing eye blinks, muscular contractions or other noise transients. Noisy electrodes were interpolated using 3D spline interpolation (Perrin et al., 1987). The ERPs were then band-pass filtered to 1–30 Hz applying the second order Butterworth low- and high-pass filters, with −12 db/octave roll-off, recalculated against the average reference, and smoothened spatially using the instantaneous spatial filter (Michel and Brunet, 2019).

The average number of accepted epochs was similar for each analyzed stimulus type (New, 58 ± 4; Rep3, 57 ± 6; N-back, 55 ± 4; ANOVA, *p* > 0.05).

### Behavioral Data Analysis

Behavioral data were analyzed using SPSS, version 20. Response accuracy in the CRT was determined as the sum of hits (correct recognition of repeated stimuli) and correct rejections of non-repeated stimuli (Table 1). The percentages of correct responses and mean response latencies were compared across New, Rep3, and N-back items in the CRT using repeated measures analysis of variance (rmANOVA) with Condition (Stimulus Type) as within-subjects factor.

**TABLE 1:** Performance in the experimental task.

	Stimulus	Response accuracy (%)	Reaction times (ms)
Continuous recognition task	*New* ^#^	97 ± 2.8	883 ± 203
	Rep1	96.9 ± 2.2	673 ± 140
	Rep2	99.7 ± 1	516 ± 117
	*Rep3* ^#^	99.9 ± 0.3	487 ± 104
	Rep4	100 ± 0	506 ± 130
	Catch	98 ± 3.5	838 ± 188
	*N-back^#^*	98.5 ± 2.1	848 ± 180
Delayed recognition (30 min.)	Novel	94.8 ± 4.5	942 ± 160
	Repeated	91.1 ± 7.3	834 ± 129
	Catch	71.6 ± 15.3	955 ± 158

*Percentage of correct responses and reaction times (ms) across different conditions in the continuous recognition task and the delayed recognition task.*

*^#^Indicates the 3 trial types of interest for the evoked potential analysis.*

### Evoked Response Potential Waveform Analysis

#### Global Waveform Analysis

The main interest of our analyses was to compare the signals induced by stimuli that -according to the logic of the study- induce encoding and recognition. To compare the differences of amplitudes between the three main trial types (New, Rep3 and N-back) we first computed electrode and time-wise ANOVAs for each of the 128 electrodes for each stimulus type. To correct for temporal autocorrelation, we only retained amplitude-differences in the cluster of minimum 10 neighboring electrodes, extending over at least 15 consecutive time points (i.e., 30 ms), with *p* < 0.005 (Guthrie and Buchwald, 1991; Murray et al., 2006; Toepel et al., 2014). *Post hoc* analyses were performed to assess the direction of main ANOVA effects. Even though ERP epoch was set to 550 ms post-stimulus onset, our main focus was the effects preceding the motor responses. As previous studies using comparable continuous recognition tasks revealed effects reflecting either recognition or encoding up to about 500–600 ms (Schnider et al., 2002; Raynal et al., 2020), we limited analysis to a window from 0 to 550 ms.

#### Fronto-Central Cluster Analysis

We then compared the amplitudes between the three main stimuli (New, Rep3 and N-back) over a fronto-central cluster of electrodes, the area in which previous studies using CRTs had shown the most important effects (James et al., 2009; Thézé et al., 2016; Raynal et al., 2020). This cluster covered the following 16 channels of Biosemi 128 channels System Layout of International 10–20 system (Jurcak et al., 2007): C1, C2, C3, C11, C12, C13, C20, Fz, C22, C23, C24, C25, C26, D1, D2, D3). Individual averaged amplitudes between the three stimulus types were compared with repeated measures ANOVA. Amplitude differences were retained as significant if they extended over at least 30 ms (15 time points) with *p* < 0.01 (Murray et al., 2006).

### Topographic Pattern Analyses

To determine periods of stable electric field topographies (“maps”), we subjected the ERPs from all 128 electrodes provoked by New, Rep3 and N-back stimuli over 550 ms post-stimulus presentation to spatiotemporal segmentation based on K-means clustering (Murray et al., 2008; Brunet et al., 2011). This method allows detecting changes in the overall pattern of the electric field (Lehmann, 1987; Murray et al., 2008), and is therefore free of the potential bias by selecting single electrodes. Modulations in strength, latency, or topographies of the template maps derived from clustering is thought to indicate differential processing across conditions (Murray et al., 2008). The optimal number of clusters (template maps) to explain the data was determined by a meta-criterion (Custo et al., 2017).

Periods of topographic stability observed in the averaged data were then compared to the original ERP data of each subject by computing the spatial correlation between each template map and each time point of individual subjects’ data (Brunet et al., 2011). This “fitting” procedure provides the measure –the Global Explained Variance (GEV)– for how well a certain template map in a given time period accounts for each individual ERP across conditions (Murray et al., 2008). The GEV values were then compared statistically with rmANOVAs. Fitting was done in time periods determined by the effects observed in the global and regional fronto-central waveform analysis and the apparent periods of stable topographies obtained with spatio-temporal segmentation.

### Source Estimation

Neural generators underlying the ERPs of each experimental condition were estimated using the Local Auto-Regressive Average (LAURA) distributed linear inverse solution (Grave de Peralta Menendez et al., 2001, 2004). The current distribution was calculated for 128 electrode positions within the gray matter of the template provided by the Montreal Neurological Institute using the LSMAC head model with a solution space of 5,000 nodes (Brunet et al., 2011). Each solution point was standardized across time in order to avoid spatial leakage and activation biases (Michel and Brunet, 2019). The estimated current densities of inverse solution points were then extracted and averaged per participant and condition in three time periods of interest: at 145–205, 210–300, and 410–470 ms following stimulus onset. These periods of interest were determined based on results of the fronto-central cluster and global ERPs, as well as on periods of stable topographies defined in topographic pattern analysis. The averaged signal over time periods of interest was then compared statistically between experimental conditions using paired *t*-tests, with *p* < 0.01, Bonferroni corrected.

## Results

### Behavioral Results

Table 1 summarizes response accuracy and reaction times across all stimulus types in the 2 recognition tasks.

Continuous recognition task: Statistical comparisons were made between stimuli of interest, i.e., those undergoing ERP analysis: New, Rep3 and N-back stimuli. Rep3 stimuli were recognized more accurately [*F*_(__2_, _38__)_ = 10.87, *p* < 0.001, *η_*p*_*^2^ = 0.364] and much faster [*F*_(__1_._55_, _29_._4__)_ = 118.81, *p* < 0.001, *η_*p*_*^2^ = 0.862] than New and N-back items. Among N-back trials, there was no difference in response accuracy or reaction times between stimuli that had previously been seen 4 times (as New, Rep1, Rep2, and Rep3) or 5 times (plus as Rep4).

In the delayed recognition task after 30 min, pictures that had been repeated in the continuous recognition task were significantly better recognized than pictures seen only once as catch trials [*F*_(__1_._41_, _26_._86__)_ = 32.93, *p* < 0.001, *η_*p*_*^2^ = 0.634]. Reaction time was faster for repeated pictures [*F*_(__2_, _38__)_ = 6.9, *p* = 0.003, *η_*p*_*^2^ = 0.267] than novel and catch items. Among repeated pictures, there was no difference between those that had been seen 5 times (as New, Rep1, Rep2, Rep3, N-back) in the continuous recognition task and those seen 6 times (plus as Rep4).

### Evoked Response Potential Waveform Analyses

#### Global Waveform Analyses

**FIGURE 2:**
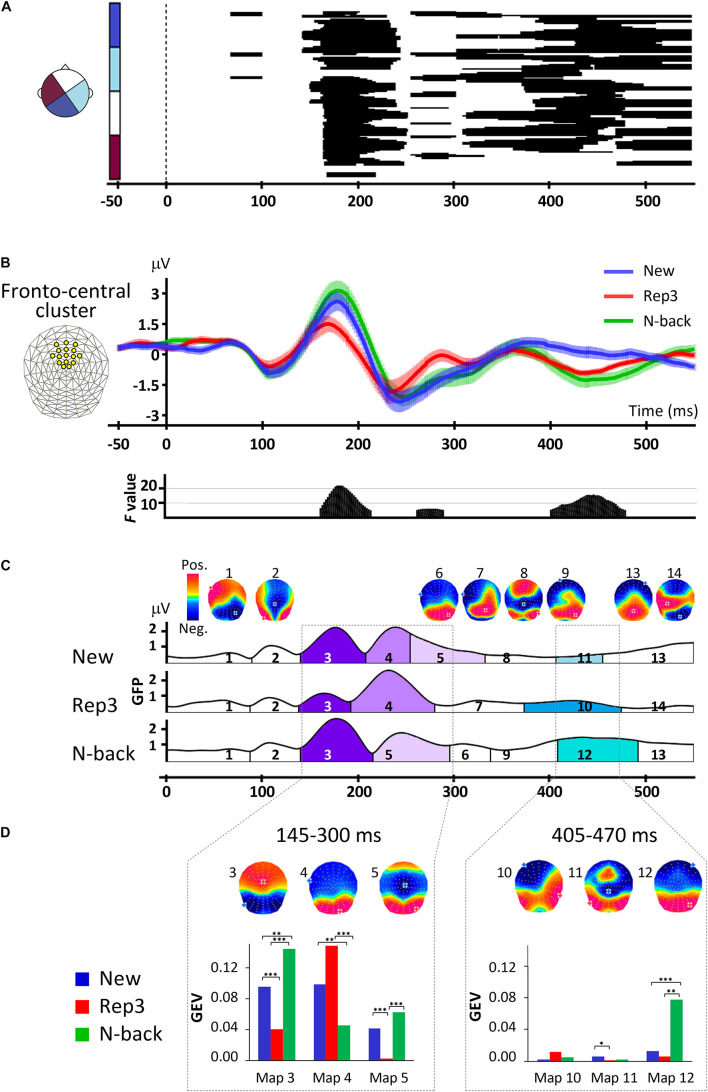
ERP analyses. **(A)** Amplitude differences over all scalp electrodes across time and conditions revealed by one-way repeated measures ANOVAs, with factor stimulus (New, Rep3, N-back). The x axis shows time (ms) and y axis displays the 128 electrodes. The black horizontal bars indicate where significant main effects occurred with *p* < 0.005, lasting for at least 30 ms. **(B)** Orienting waveforms analysis over a fronto-central cluster of 16 electrodes indicated on the upper left. The lower panel displays the *F*-value of the main effects of one-way repeated measures ANOVA over the cluster with the factor of stimulus type (New, Rep3, N-back) revealing three periods of significant amplitude differences. **(C)** Spatiotemporal analysis. Temporal distribution of stable electric field topographies in New, Rep3 and N-back conditions and their corresponding cortical maps. The curves indicate the GFP (Global field power). **(D)** Statistics of fitting the group-averaged topographies to single subject ERPs. Bar graphs display the GEV (Global Explained Variance), the measure of how well a given map in a given period of time represents individual data. ^∗^*p* < 0.05, ^∗∗^*p* < 0.01, ^∗∗∗^*p* < 0.005.

Figure 2A presents the result of a one-way ANOVAs with the 3 trial types (New, Rep3, N-back) over all scalp electrodes. It reveals multiple, extended periods of significant effects of condition (trial type) expressed over varying combinations of electrodes (black areas in Figure 2A).

In a first period at about 150–250 ms, differences extended over almost all electrodes. *Post hoc* tests showed that in this period, Rep3 stimuli differed from both New and N-back stimuli.

A second period with more delicate differences expressed over few electrodes appeared at 260–290 ms, followed by differences over varying electrode combinations from about 300–370, 370–450, and 430–550 ms. Over this whole period (300–490 ms), responses to New and N-back differed, with strongest difference over extended scalp areas occurring at around 390–480 ms.

#### Fronto-Central Cluster Analyses

Periods of amplitude differences were sharply delineated in the fronto-central cluster ERPs As evident in Figure 2B, the traces started to separate at about 160 ms post-stimulus onset, with N-back and New items inducing a more positive wave than Rep3 items. Then, from 260 ms Rep3 items induced a more positive amplitude than New and N-back items. At 405–480 ms, N-back (and Rep3) items induced a more negative potential than New items. [Given the short reaction times (487 ± 104 ms), the potential evoked by Rep3 may, however, be contaminated by motor preparation, which sets in about 100 ms before motor execution (Thorpe and Fabre-Thorpe, 2001)]. The time periods when the main effects occurred (time-wise rmANOVA *p* < 0.01, duration ≥ 30 ms) were 160–215, 260–290, and 405–480 ms after stimulus onset.

### Topographic Pattern Analyses

The spatiotemporal segmentation applied to New, Rep3 and N-back ERPs, yielded 14 dominant template maps that described 93% of the whole dataset. Figure 2C displays the temporal distribution of these template maps. This illustration indicates the dominant map at a given point in time over the whole group of subjects; the amplitude of the curve indicates the Global Field Power (GFP), a measure for the strength of the electric fields. Figure 2D gives the statistical analysis of the comparison of the Global Explained Variance (GEV) of the maps, a measure for how well a certain map accounts for each individual’s ERP across conditions (Murray et al., 2008).

Based on the effects observed in the global and fronto-central waveform analysis and the apparent periods of stable topographies obtained with spatio-temporal segmentation, fitting was done in the following time windows: a first period combining the two adjacent early periods observed in the waveform analysis (145–205 ms, 210–300 ms), that is, 145–300 ms, and late period from 405 to 470 ms.

From 145 to 300 ms, there was a significant interaction of Map [maps 3, 4, 5] × Condition [*F*_(__2_._8_, _53_._2__)_ = 21.88, *p* < 0.001, *η_*p*_*^2^ = 0.54], and a significant effect of Map [*F*_(__1_._54_, _29_._2__)_ = 5.88, *p* < 0.01, *η_*p*_*^2^ = 0.24]. *Post hoc* analyses showed that Maps 3 and 5 had a stronger GEV in response to N-back and New than Rep3. Map 4 had a stronger GEV in response to Rep3 and New than N-back. Thus, two of the maps (3 and 5) have stronger expression in response to New and N-back stimuli than to Rep3.

In 295–405 ms period there was no significant Map [maps 6, 7, 8, 9] × Condition interaction (*p* > 0.05).

At 405–470 ms, there was a significant interaction of Maps [map 10, 11, 12] × Condition [*F*_(__1_._37_, _26_._08__)_ = 14.62, *p* < 0.001, *η_*p*_*^2^ = 0.44], and a main effect of Condition [*F*_(__1_._27_, _24_._19__)_ = 18.39, *p* < 0.001, *η_*p*_*^2^ = 0.49] and Map [*F*_(__1_._15_, _21_._75__)_ = 12.35, *p* < 0.001, *η_*p*_*^2^ = 0.39]. This was due to a stronger Map 12 in response to N-back than New and Rep3. (In view of the short reaction time, the response to Rep3 in this time period has to be taken with caution due to probable influences of response preparation).

### Source Estimation

Source estimation was performed for 3 time periods determined by the waveform analysis, underscored by differences of map expression in these periods: 145–205, 210–300, 405–470 ms.

**FIGURE 3:**
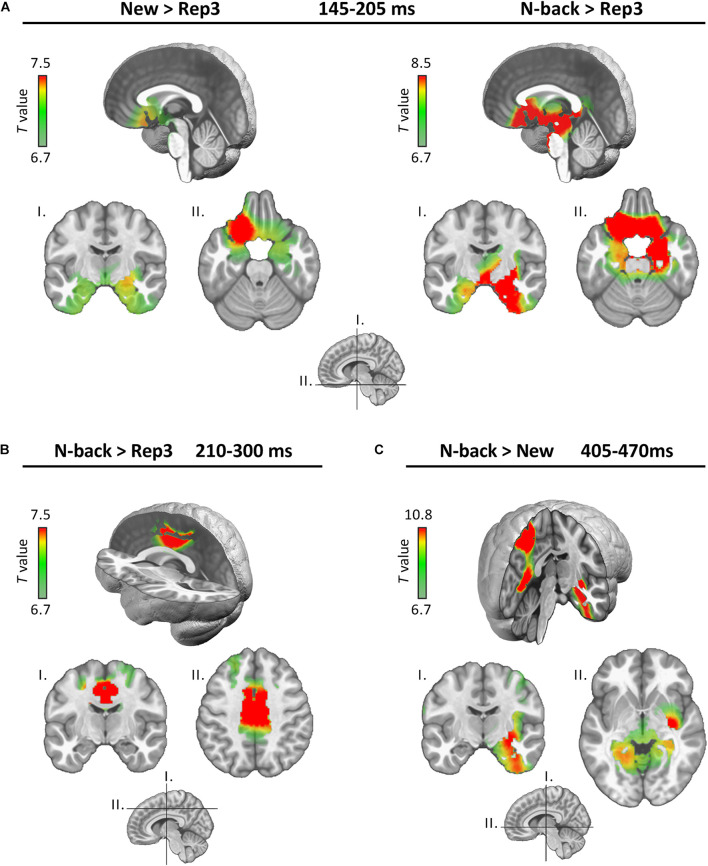
Source estimations. Estimated neural generators emerging from the indicated comparisons in the three periods (**A:** 145–205 ms, **B:** 210–300 ms, and **C:** 405–470 ms). Only significant differences are displayed.

Figure 3 displays the comparisons of brain activations in the three periods of interest.

In the 145–205 ms period (encoding), both N-back and New stimuli activated the MTL (particularly on the right) and the posterior orbitofrontal cortex (OFC) more strongly than Rep3 (Figure 3A). Comparisons between New and N-back conditions yielded no significant difference in this time period.

In the subsequent 210–300 ms period (Figure 3B, continued encoding plus expression of a map in response to Rep3), N-Back stimuli activated the dorsal anterior cingulate (dACC) and the middle cingulate (MCC) cortex more strongly than Rep3 stimuli. New did not significantly differ from Rep3, or from N-back. Rep3 did not induce stronger activation than New or N-back in any region. This period may correspond to continued encoding which achieved significantly different intensity of activation only in the comparison of N-Back and Rep3.

At 405–470 ms (Figure 3C), N-Back, in comparison to New items, induced greater activation in right MTL, insular and restrosplenial cortex (including precuneus) and parietal areas than New stimuli. Other comparisons (New vs. Rep3, N-back vs. Rep3) yielded no significantly different activations.

## Discussion

This study indicates temporally distinct memory processes occurring very rapidly after presentation of visual stimuli, whereby an encoding signal (around 150–300 ms) appears to precede recognition (≥ 400 ms). The MTL is involved in both processes.

The study relied on three essentially independent types of analyses: an orienting global and fronto-central cluster waveform analysis; a spatiotemporal analysis to determine differences in the electrocortical potential patterns (“maps”) of the whole set of 128 electrodes (Lehmann, 1987; Murray et al., 2008; Brunet et al., 2011); and source estimation using inverse solutions (Grave de Peralta Menendez et al., 2001, 2004; Michel and Brunet, 2019).

Encoding and recognition were planned to be extracted by comparing the responses to different stimuli, based on the following assumptions: (1) Stimuli appearing for the first time in a continuous recognition task (“New”) undergo encoding, as revealed by their recognition after multiple intervening stimuli (Schnider et al., 2002; Wahlen et al., 2011) and 30 min after completion of the task (James et al., 2009). In the present study, stimuli presented only once in the continuous recognition task (catch trials) were well, albeit not perfectly, recognized 30 min after termination of the task. (2) Items appearing after intervening items, i.e., N-back items, differ from their first appearance as “New” items by the fact that they can be recognized as having been seen before. In the present study, too, items recurring as N-back were recognized almost perfectly. In addition, stimuli that are repeated after a delay, that is, after multiple intervening items (N-back in the present study), are later also better recognized than stimuli presented only once (James et al., 2009). Thus, they obviously also undergo new encoding. (The present study was not designed to replicate this finding; N-back trials constituted the fifth or sixth presentation of stimuli and were then much better recognized after 30 min than stimuli seen only once). (3) Stimuli repeated 3 times after initial presentation (Rep3) were used as a “baseline.” Repeated presentation of the same stimuli engages less neural resources, a processing advantage called repetition suppression (Brozinsky et al., 2005; Grill-Spector et al., 2006; Johnson et al., 2008). In the present study, this effect was very strong: reaction times decreased more than 40% for these stimuli. As evident in Table 1, stimuli were treated with similar efficacy when they were immediately repeated for the second (Rep2), third (Rep3), or –for half of the stimuli– for the fourth time (Rep4). Also, upon re-appearance as N-back stimuli or in the 30-min delayed recognition task, stimuli that had been immediately repeated only 3 times were as accurately and rapidly recognized as stimuli that had been repeated 4 times. Thus, these late repetitions did not significantly increase retention, and thus, encoding, of the stimuli. In the source estimations, Rep3 trials did not evoke stronger activation in any brain region than the New or N-back stimuli. These observations support these stimuli’s use as minimal “baseline activity” for discerning processes common to New and late-repeated stimuli (N-back), in particular encoding.

A caveat concerning these assumed processes is that New and N-back stimuli differ from the preceding stimulus, while Rep3 stimuli are repetitions of themselves. Thus, apart from encoding, New and N-back stimuli probably also induce perceptual processes (e.g., attribution of meaning to the new stimulus) and heighten attention beyond the reactions to Rep3 trials. Indeed, it might be impossible to reliably disentangle encoding, perception, and attention. Following a prominent theory, memory traces are formed by the stabilization (under the influence of the MTL-hippocampus) of the neural networks originally processing a piece of information (Fuster, 1995) –perceptual processing and encoding may go hand in hand. Our findings support this idea and suggest that New and N-back stimuli differ from Rep3 by more than encoding.

New and N-back stimuli differed from Rep3 trials in an early period, between 145 and 300 ms, as deduced from a different fronto-central waveform (Figure 2B) and different map configurations (Figures 2C,D) of New and N-back trials in comparison with Rep3 trials. This finding is consistent with an earlier study showing ERP differences between New and 4-times repeated stimuli at about 180–300 ms (Raynal et al., 2020). Paradigms using separate learning sessions typically reported later encoding phases starting at about 250 ms (Ranganath and Rainer, 2003; Patel and Azzam, 2005).

This period appeared to encompass two different stages, as already suggested by the waveform analysis. Source localization indicated stronger MTL and orbitofrontal activation in response to New and N-back stimuli in comparison to Rep3 only in the initial phase, from 145 to 205 ms (Figure 3A). Thus, even if one speculated that this early processing encompassed perceptual and attentional processes in addition to encoding, it is noteworthy that it was dominated by limbic activity (MTL, orbitofrontal). Although source localization with high-density EEG does not have high spatial resolution, recent studies showed that it is able to seize activity in subcortical and medial temporal structures (Nahum et al., 2011; Seeber et al., 2019). Given the well-known implication of the MTL in memory encoding (Squire et al., 2004; Kim, 2015; Moscovitch et al., 2016), this activation is likely to reflect an encoding signal.

In the second phase, at 210–300 ms, both New and N-back stimuli induced a different amplitude and expressed a specific map more strongly than Rep3 (Figures 2C,D; map 5); inverse solution indicated stronger activation in response to N-back than Rep3 stimuli (Figure 3B). This activation concerned the middle cingulate cortex, an area that has been linked to multiple functions, including decision making and attention (Bush et al., 2002; Apps et al., 2013; Vogt, 2016), set-shifting (at 200–300 ms) (Perianez et al., 2004) and working memory (Petit et al., 1998). Notably, an fMRI study using a prospective memory task on place-object associations described modulations of the MTL by the dorsal anterior cingulate during encoding, which was predictive of learning proficiency (Woodcock et al., 2015). If the assumptions regarding the processes evoked by the different stimuli in the present study are correct, this processing difference may reflect encoding of New and N-back stimuli, in concert with other, in particular attentional, processes.

At the beginning of this period, Rep3 stimuli also expressed a specific map more strongly than New and N-back stimuli (Figures 2C,D, map 4). A possible interpretation of this map is perceptual priming, thought to account for improved performance in repetition suppression (Maccotta and Buckner, 2004; Schacter et al., 2007). This interpretation is compatible with the observation that Rep3 stimuli did not induce a demonstrably stronger activation of any brain area in this period in the inverse solution than the other stimuli.

Recognition, as determined by the contrast between N-back and New stimuli, induced differences in the waveform amplitude (Figure 2B) and the topographic pattern analysis (expression of map 12 in Figures 2C,D) at about 400–500 ms. The timing is comparable to other studies exploring old/new effects (Friedman, 1990; Schnider et al., 2002; Rugg and Curran, 2007; Addante et al., 2012). Source localization demonstrated stronger activation in the MTL, insula, retrosplenial cortex and parietal cortex in response to N-back than New items. The implication of the MTL in recognition was expected (Lepage et al., 1998; Zeineh et al., 2003; Kim, 2015). The activation of the areas beyond the MTL is more intriguing. These areas correspond to a network that has been suggested to be involved in recollection (actual remembering of the stimulus presentation) rather than detection of familiarity (Rugg and Vilberg, 2013). It is plausible that the initial presentation of stimuli followed by multiple repetitions allowed for an event to be encoded that was amenable to recollection as a stimulus previously seen in the present task.

The conclusion that an encoding process, in association with preceptual and attentional processes (145–300 ms), precedes a recognition signal (405–470 ms) has important implications.

If one assumes that the encoding signal initiates –rather than delimits– an encoding process, a logical consequence would be that all that happens to the memory trace from its initiation or re-activation until (or beyond) its recognition will be encoded. This sequence may be important for diverse memory and thought processes. One instance was mentioned in the introduction: Orbitofrontal reality filtering (ORFi), the capacity to synchronize thought and behavior with ongoing reality within –or despite– a free flow of thoughts (Schnider, 2013, 2018). Failure of this mechanism is associated with reality confusion as reflected in confabulations, disorientation and acts that do not relate to current reality (Schnider and Ptak, 1999). ORFi is electrophysiologically expressed at 200–300 ms after stimulus presentation (Schnider et al., 2002; Wahlen et al., 2011; Bouzerda-Wahlen et al., 2015) and thus precedes recognition of the memory’s content, which –as again suggested by the present study– occurs after 400 ms. A previous study suggested that the ORFi signal is preceded by an encoding signal by approximately 30 ms (Thézé et al., 2017). The present study indicates that the encoding signal may occur even earlier. This sequence of processes would thus leave a memory trace that contains information about whether one has actually experienced a certain situation or only thought about it –the essence of source monitoring (Johnson et al., 1993; Mitchell and Johnson, 2009; Thézé et al., 2017).

A final implication concerns the MTL’s role in memory consolidation. While the standard consolidation theory holds that consolidation is a prolonged one-time event (Squire et al., 2015), alternative views posit that consolidation happens through reactivation and re-encoding of memories (Nadel et al., 2000; Dudai et al., 2015). The duration of MTL influence after encountering a piece of information is unclear. Evidence from fMRI studies (Dudai et al., 2015) suggest at least seconds (given the temporal resolution of fMRI). Continuing hippocampal-neocortical interaction was even demonstrated for minutes after an event (Tambini et al., 2010; van Kesteren et al., 2010). Whatever the precise duration of MTL involvement in consolidation, the results of this study, as well as the earlier studies (James et al., 2009; Raynal et al., 2020), indicate that –at least in this early phase of stimulus processing– the MTL activity allowing for encoding is phasic rather than tonic, lasting less than 100 ms at a time. One may speculate that the hippocampus provides a brief signal to cortical areas synchronously processing a piece of information at a given moment, allowing them to establish networks according to Hebbian rules (Fuster, 1995). This idea is compatible with the recent description of memory encoding (and retrieval) being accomplished by a delicate, finely tuned interplay of neural ensembles in the MTL and cortical areas (Vaz et al., 2020). This MTL-neocortical interaction may repeat every time a memory is re-activated and re-encoded, finally leading to consolidation, as proposed by the Multiple Trace Theory (Nadel et al., 2000).

## Data Availability Statement

The datasets presented in this article are not readily available because the participants’ consent did not cover public data sharing. Study data can be obtained from the corresponding author upon reasonable request and on the condition of authorization by the Ethical Committee.

## Ethics Statement

The studies involving human participants were reviewed and approved by the Ethical Committee of the Canton of Geneva, Switzerland. The patients/participants provided their written informed consent to participate in this study.

## Author Contributions

DT: conception of the research, protocol design, data collection, data analysis, writing, reviewing, and editing of the manuscript. AA-D: data collection, supervision, reviewing, and editing of the manuscript. RP: reviewing and editing of the manuscript. AM: conception of the research, supervision, reviewing, and editing of the manuscript. AS: conception of the research, funding acquisition, protocol design, supervision, writing, reviewing, and editing of the manuscript. All authors have made a substantial contribution to the work, and approved the submitted version of the article.

## Conflict of Interest

The authors declare that the research was conducted in the absence of any commercial or financial relationships that could be construed as a potential conflict of interest.

## Publisher’s Note

All claims expressed in this article are solely those of the authors and do not necessarily represent those of their affiliated organizations, or those of the publisher, the editors and the reviewers. Any product that may be evaluated in this article, or claim that may be made by its manufacturer, is not guaranteed or endorsed by the publisher.

## Footnotes

1
https://doi.org/10.26037/yareta:42i5fnhkjfhotaxfyhtqqtdbbu

2
https://sites.google.com/site/cartoolcommunity/

3
http://doi.org/10.5281/zenodo.1164038
